# Therapeutical Hints

**Published:** 1886-03

**Authors:** 


### Therapeutical Hints.

Dr. John W. Martin says in the *Med. Press*, July 22, that the following is an excellent lotion for subduing inflammation, and reducing the œdema of the inflamed parts:

| | | | |
|---|---|---|---|
| ℞. | Tr. opii camph. co., | ʒij. | |
| | Tr. tolutani, | ℥ij. | |
| | Liq. plumbi diacetat, | ℥iv. | |
| | Glycerine, | ʒij. | |
| | Aquæ, ad., | ℥xx. | M. |

A piece of lint or old linen to be well wetted with the lotion, and to be applied to the inflamed part. The wetting to be repeated at frequent intervals.

Internally, it is useful to combine the following mixture with the use of the foregoing lotion:

| | | | |
|---|---|---|---|
| ℞. | Potass bicarb., | ʒiss. | |
| | Tr. nucis vom., | mxl. | |
| | Ferri am. cit., | ʒiss. | |
| | Sp. am. aromat., | ʒiss. | |
| | Aquæ, ad., | ℥viij. | Liq. M. |

" ʒj. three or four times a day.

He has found this treatment especially useful in those cases in which intense inflammation in the arms follows re-vaccination.

